# Reducing the anxiety of patients undergoing an oral biopsy by means of graphic novels: an open-label randomized clinical trial

**DOI:** 10.4317/medoral.25950

**Published:** 2023-06-18

**Authors:** Monica Bazzano, Rodolfo Mauceri, Giulia Marcon, Giuseppina Campisi

**Affiliations:** 1 Department of Surgical, Oncological and Oral Sciences (Di.Chir.On.S.), University of Palermo, Palermo, Italy; 2 Department of Engineering, University of Palermo, Viale delle Scienze, Palermo, Italy; 3 Oral Medicine and Dentistry Unit for frail patients, Department of Rehabilitation and continuity of care, University Hospital Palermo, Palermo, Italy

## Abstract

**Background:**

The use of graphic novels is a trending topic in health communication as a new form of visual storytelling which explores narratives of health care, cancer, healing, and disability. The objective of the present study was to evaluate - for the first time in the literature - the effect of graphic novels in reducing the anxiety of patients waiting for an incisional biopsy in an oral oncology setting.

**Material and Methods:**

This open-label randomized clinical trial comprised 50 patients with a clinical suspicion of oral potentially malignant disorders. Twenty-five patients were randomly allocated to the test group, and a colourful graphic novel was provided. Subsequently, the Beck Depression Inventory and the Depression Anxiety Stress Scales-21 were administered to all 50 recruited patients, after which a biopsy was performed on each patient.

**Results:**

No statistically significant difference was observed between the test and control groups for the variables regarding the demographic data (*p*>0.2). There was a significant difference after the introduction of the graphic novel, regardless of which questionnaire was used. The graphic novel demonstrated an improvement in the ability of the test group to tolerate anxiety while waiting for an oral biopsy in both psychological tests (*p*<0.05).

**Conclusions:**

In light of these initial positive results, the authors of this study would like to suggest the use of graphic novels in oral oncology, dentistry, and medicine with the aim of reducing patient anxiety.

** Key words:**Graphic novels, oral medicine, oral biopsy, potentially malignant disorders, oral cancer, anxiety, phobia.

## Introduction

Oral medicine is the main speciality, together with maxillofacial surgery, dealing with severe and on occasions life-threatening orofacial disorders or outcomes; it, therefore, elicits states of fear and anxiety in patients (1). A phobia can be defined as a persistent tendency to respond with increased arousal towards a specific object or situation, comprising features of rationality. The fear and/or anxiety related to phobia can be said to be disproportionate when compared to the actual risks or objective threat of the situation to be faced, such as an oral biopsy (2).

Patients with a specific phobia often experience intense and immediate fear and/or anxiety prior to dental treatment and/or an oral biopsy. Their psychological state of stress and discomfort usually develops when they are in the waiting room, to possibly include negative intrusive thoughts (e.g., "it will hurt, I will be diagnosed with a terrible disease")(3). Whilst often recognizing that their fear or anxiety is excessive, these patients experience prolonged states of anxiety to the point of avoiding phobic situations (4). Waiting for a biopsy can be fraught with fear: "What if it is cancer?" Patients tell us and teach us that, in the grip of fear, we learn to arm wrestle between "it could be yes" and "it could be not". Indeed, a cancer diagnosis is probably one of the most stressful, life-changing events (5).

The term graphic medicine was coined by a group of researchers, clinicians, and artists with the aim of exploring this new subfield of research and practice (6). It can be defined as a form of visual storytelling exploring narratives of health care, cancer, healing, and disability (7). Graphic medicine is currently trending topic in health communication, as messages, which are contained within the storytelling of health communication, are increasingly described as “creative” and “compelling” (8).

Researchers are becoming increasingly interested in graphic narratives, with which to convey persuasive public health messages (e.g., Centers for Disease Control and Prevention, 2011). Narratives with comics also continue to emerge as effective communication tools of public health (9,10). Various authors theorize the role of storytelling with comics in shaping behaviours, as in the case of anxiety reduction. Graphic medicine is considered effective by many practitioners because it offers the opportunity to provide medical professionals with an innovative approach to information, which is rapid and comprehensive (11).

The visual narratives which can be considered most relevant to the study of graphic medicine are comic books in their various guises. Graphic medicine examines the use of sequential (but not always narratively linear) visual storytelling, with which to share health-related experiences or information (12). The use of comics, therefore, facilitates the construction of soft skills, thereby opening up a space in which care professionals can pause and reflect as to how to communicate effectively with patients. With their combination of images, limited text, and relatively short format, comics provide an option for the effective communication of health-related messages (13).

Consequently, graphic novels have been applied to several fields, including: oncology (14), HIV infection (15), and eating disorders (16). To date, there have been few applications in the field of dentistry, with the exception of paediatric dentistry, which has yielded positive results (17).

The objective of the present study was to evaluate - for the first time in the literature - the efficacy of colourful graphic novels in reducing the anxiety of adult patients waiting for an incisional biopsy in an oral oncology setting.

## Material and Methods

- Study design

This open-label randomized clinical trial (RCT) was approved by the local Institutional Ethics Committee of the P. Giaccone University Hospital of Palermo, Italy (approval #1/2022). The study was conducted according to the principles of the Declaration of Helsinki regarding experimentation involving human subjects, and written informed consent was obtained from all participants.

The authors consecutively included all patients with a clinical suspicion of an oral potentially malignant disorder (OPMD), who had been referred to the Oral Medicine Unit at the P. Giaccone University Hospital in Palermo (Italy), from May to November 2022. Prior to the oral incisional biopsy, 50 patients were randomly allocated (i.e., computer-generated) to the test or control group; a graphic novel was provided to 25 patients in the test group.

- Eligibility criteria

1. age of patients ≥ 18 years

2. absence of suspected or obvious pregnancy status in female subjects

3. patients affected by OPMD

4. agree to participate in the RCT, and the ability to read and understand informed consent

5. patients not affected by any known psychological disorder.

- Exclusion criteria

1. patients with cognitive deficits, such that they cannot adequately complete the questionnaire and the visually impaired

2. patients who are to undergo surgical treatment and/or biopsy, for reasons other than OPMD (e.g., salivary gland biopsy)

3. patients using benzodiazepines or other psychotropic medications.

- Oral graphic novel description

**Figure 1:**
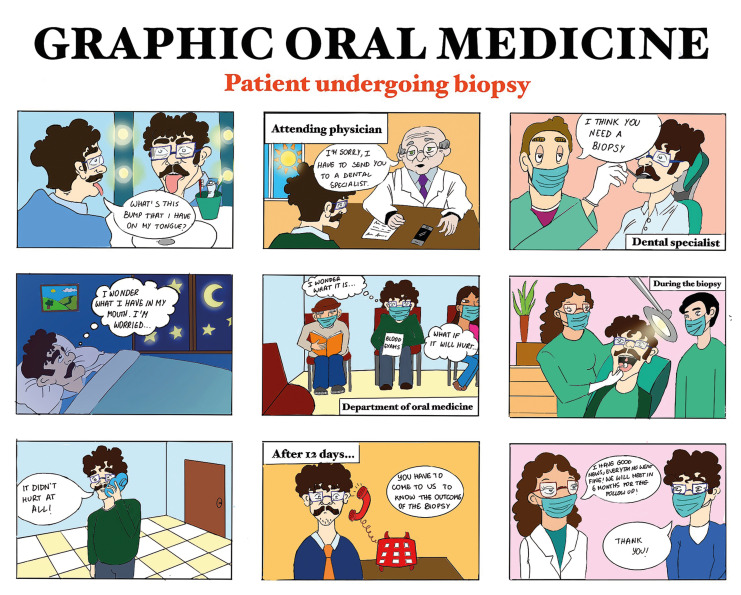
The English version of the graphic novel, offered to the test group (with thanks to G. Piazza).

The authors used a comic strip consisting of a sequence of 9 colourful vignettes, in which the procedure of an oral biopsy was described to the patient. (Fig. 1). The authors paid close attention to the facial expressions of the characters and the background (e.g., tone of the scene), both similar to the effective action contexts experienced by patients in the Oral Medicine Unit. The vignettes are described in detail below from left to right:

1. A patient in the mirror is inspecting their oral cavity and they find something abnormal

2. The patient seeks the clinical opinion of a physician

3. The patient is examined by their dentist

4. Thereafter, during the night, the patient ruminates as to what this abnormality might be

5. Having been referred for diagnostic procedures, the patient is in the waiting room of the Oral Medicine Unit

6. The Oral Medicine team greets the patient, and performs an OPMD biopsy

7. Post-biopsy, the patient is leaving the Oral Medicine Unit while applying an ice pack after surgery

8. The patient is requested (via a phone call) to return for the histology results

9. An oral physician informs the patient of the histological findings.

- Description of tests used to assess anxiety

All the 50 recruited patients received the Beck Depression Inventory (BDI) and the Depression Anxiety Stress Scales-21 (DASS-21). Prior to the surgical procedures (i.e., oral biopsy), these questionnaires were administered anonymously in a mixed paper-pencil and QR Code mode. The administering psychologist (M.B.) was present for questions and clarification. Before filling out the BDI and DASS-21 questionnaires, patients were asked to answer some questions regarding their demographic data and medical history (e.g., smoking habits). The BDI is a self-administered instrument for assessing the severity of the affective, cognitive and motivational psychomotor, and the vegetative components of depression. It comprises 21 sets of statements, describing various and increasing levels of depressive symptomatology; each statement corresponds to a score, and these scores are totalled to produce a total score. Higher scores of BDI are correlated with major depressive state (18).

The present study also used the psychometric properties of the Italian version of the DASS-21 to explore its use in an Italian context. The DASS-21 facilitates the identification of three constructs: depression, anxiety, and stress. Depression includes: dysphoria, hopelessness, devaluation of life, lack of interest/involvement in regulating daily life, anhedonia, and inertia. Anxiety relates to: autonomic nervous system arousal, skeletal muscle effects, situational anxiety, and the subjective experience of anxious effects. Stress relates to: the presence of chronic non-specific arousal levels, relaxation difficulties, nervous arousal, irritability, agitation, hyperactivity, and impatience (19).

A digital platform, including the following, was created:

1. a section with two separate BDI, DASS-21 tests/forms for patients, who completed them anonymously. And, in order to enhance the information collating process, a QR code was created for each test/form in such a way that patients could easily scan these with their cell phone camera and quickly complete each test/form

2. an administrative section, including a Dashboard (providing an overview of the tests/forms, to be completed daily) and several subsections permitting the extrapolation of statistical data, each form being based on the answers provided by the patients. The React framework (for the front-end) and Php and MySql (for the back-end) was used to create the web platform.

- Outcome Measures

The following data were recorded for each patient: demographic data, smoking habit, drug intake, the BDI scores according to the An Inventory for Measuring Depression (18), and the DASS-21 scores according to the Italian version (20).

- Statistical Analysis

The analysis was performed using the software R (R Core Team, 2022). Descriptive statistics were used to analyse the patients’ socio-demographic and physical characteristics. BDI and DASS-21 scores were summarized through mean value, standard deviation (SD), maximum, median and interquartile range (IQR). In order to analyse any significant statistical difference of anxiety between the test and control groups, the data were initially tested to ascertain if any socio-demographic or physical characteristics were associated with group membership by Chi-Square Test. The Mann-Whitney U test was used to evaluate the significant differences between treatment groups, with respect to BDI and DASS-21 scores. In order to quantify any statistically significant difference, the difference between BDI and DASS-21 scores in the groups was evaluated by the Hodges-Lehmann estimator. Any relation between BDI and DASS-21 scores was measured through a linear correlation coefficient.

## Results

**Figure 2:**
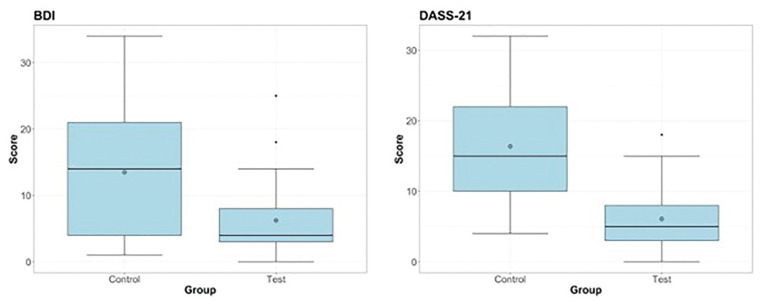
Boxplot describing the distribution and summary statistics of BDI and DASS-21 scores for the control and test groups.

The descriptive statistics relating to the patients’ socio-demographic and physical characteristics are displayed in Table 1. No statistically significant difference was observed between the test and control groups for the variables regarding the demographic data. Since all *p-value*s were greater than the chosen significance level (0.05), this would ensure that any difference in the BDI and DASS-21 scores were unrelated to the aforementioned characteristics of the two groups. Summary statistics relating to the BDI and DASS-21 scores, overall and corresponding to both test and control groups, are displayed in Table 2, while the corresponding distributions are presented in Fig. 2.

**Table 1:**
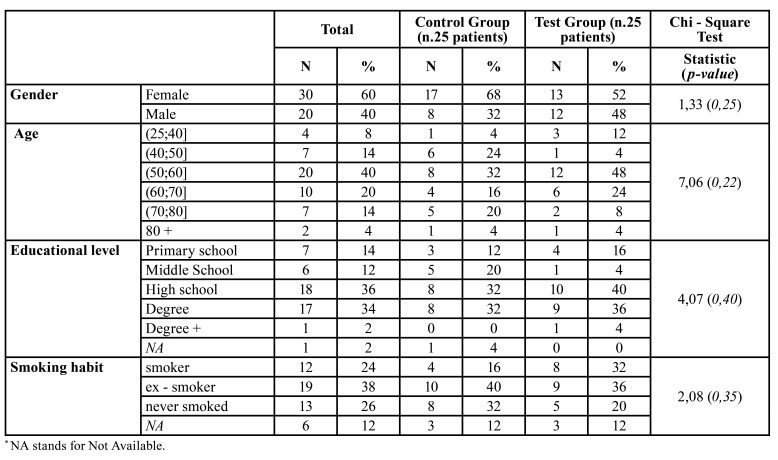
Descriptive statistics relating to patient socio-demographic and physical characteristics.

**Table 2:**
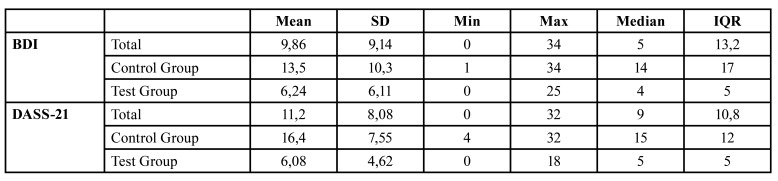
Summary statistics of the BDI and DASS-21 scores.

The results are shown in Table 3 and Table 4: the two-sided test *p-value*s highlighted in bold, conducted on the total scores, were both lower than 0.05, thereby demonstrating that there was a significant difference when offering the graphic novel to the patient, regardless of which questionnaire was used. Moreover, the Hodges-Lehmann estimator predicted a reduction of approximately 5 points in the median BDI score when the graphic novel was offered to patients prior to their surgical procedures. Similarly, the difference in DASS-21 scores between the study groups was found to be statistically significant, with the Hodges-Lehmann estimator suggesting that this difference approximate to 10.

Each question of the BDI and DASS-21 questionnaires was also tested for differences between treatment groups. Table 3 and Table 4 display these results: specifically, the columns on the far right contain the results of the Mann-Whitney U test and corresponding *p-value*. Both BDI and DASS-21 questionnaires displayed significant differences with respect to crying, loss of interest in regulating daily life, indecisiveness (BDI) and anxiety (DASS-21).

Thus far, a significant difference in BDI and DASS-21 scores has been observed between the test and control groups respectively. Indeed, a strong positive correlation was observed between the BDI and DASS-21 scores. The linear correlation coefficient generally equalled 0.78 (*p-value* = 2.15938e-11). Similarly, the linear correlation coefficients in the test and control groups, 0.74 and 0.76 respectively, were found to be moderately strong.

**Table 3:**
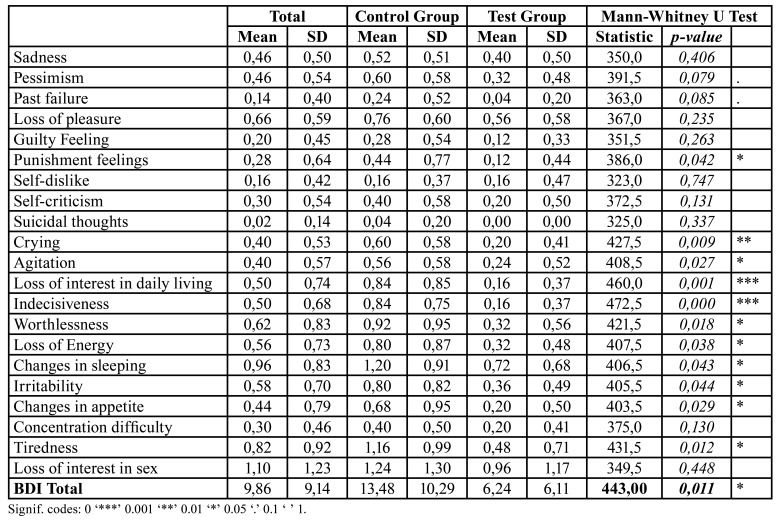
Mean and standard deviation, and Mann-Whitney U Test for each BDI question and total score.

**Table 4:**
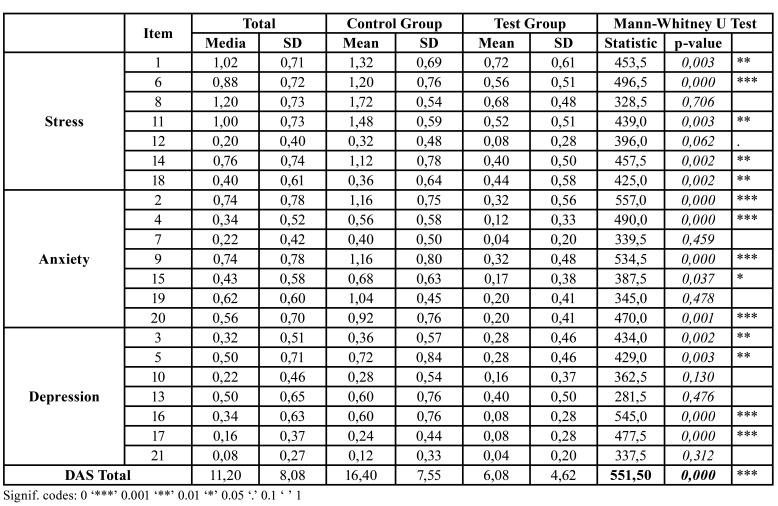
Mean and standard deviation, and Mann-Whitney U Test for each DASS-21 question and total score.

## Discussion

This study has proposed for the first time in the field of dentistry the use of a graphic novel, which is aimed at adults, that is, patients who are waiting for an incisional biopsy to ascertain the presence of OPMD. A further component of the study is to assesses its efficacy in reducing patient anxiety, as measured by BDI and DASS 21scores. It was observed that patients, who were offered a comic book while waiting to undergo an incisional biopsy were more informed about this subject than those who did not. They consequently experienced less anxiety (also in accordance with the theory of reasoned action) (21).

There is an increasing need for new tools in the field of healthcare, with which to develop strategies of effective communication in medicine and dentistry. These include; the use of colour and ludic activities, infographic videos or leaflets (22). It is imperative that medical practitioners constantly seek effective ways of talking with patients (e.g., narrative medicine), and identify training techniques which teach healthcare professionals to consider the emotions and interpersonal skills of the patients (6). Many consider that graphic medicine provides a new, information-based approach for healthcare professionals, which is also rapid and comprehensive (11). Numerous studies have demonstrated how the creation of comics and visual narratives can improve the communication activities between clinicians, patients, and caregivers, even in cases involving medical information, using, for example, photos, illustrations, and graphic visualizations (23). To date, positive results have been observed paediatric dentistry (17).

It has been demonstrated that combining pictures and text improves comprehension because reading and viewing activate different information-processing systems within the brain (24). For example, Cruz *et al*. have created a colourful comic strip, which was appreciated by the reader, who responded positively to the narrative through colourful drawings (25). With a few pictures, it is possible to deliver extremely effective health education messages to patients, the information contained therein therefore empowering patients. By means of the graphic novel, it is also possible to convey the patients’ emotional condition and challenges to the doctor and/or caregivers. Indeed, the use of comics seems to facilitate the construction of soft skills, thereby opening up a space in which care professionals can pause and reflect as to how to communicate effectively with patients.

In oral medicine, the dread of undergoing a biopsy is significant, leading to an unpleasant emotional state, which can affect the way a patient feels, thinks, and acts. Feelings of distress operate along a continuum, ranging from commonly-shared feelings (such as vulnerability, sadness, anger, and fear) to challenges which can quickly evolve into mental disorders (e.g., depression, anxiety, panic), social isolation, and existential and spiritual crises. The distress generated by the fear of having cancer typically negatively impacts a patient’s life, and it, therefore, plays a key role in diagnostic delay and assisting a patient to return to their before-the cancer diagnosis life (26,27).

Such conditions of distress are strictly related to cognitive-behavioural theory, which considers emotions and behaviour to be closely intertwined. Specifically, thoughts can be said to influence emotions and behaviour, thus it is not the actual event which causes psychological distress (i.e., the oral biopsy), but one’s perception of that event. This implies that patients’ emotional and behavioural reactions can be determined by the way in which they interpret different situations and the attributed meaning of those events (2).

The genre of the colourful graphic novel has been adapted to the field of oral medicine, after which it was necessary to evaluate its efficacy in reducing patient anxiety as they waiting for an incisional biopsy in presence of a possible OPMD.

The results of the present study seem to confirm that the application of graphic medicine to a psychoeducational approach alleviates anxiety and distress of patients. In turn, this leads to a more accurate understanding of the risk, while facilitating communication with the patient. This study has provided an example of how this approach works and how it could be integrated into medical education and dental practice.

- Limitations

This RCT has been based on a small group of patients, and the context of administering the comic book reading and test completion is not considered to be particularly appropriate. On occasions, the target reader may not identify with the comic book character because the setting does not reflect their typical habits, expressions, type of language, and other aspects related to daily life. Regrettably, it was not possible to perform a blind RCT.

## Conclusions

Comics can be said to communicate concepts by telling stories with relevant characters, settings, and situations; they are also accessible, even for patients with different educational levels. Graphic novels are a recently-created, original and creative way of informing patients about diseases or medical procedures. In the scenario outlined in this paper, a graphic novel has been demonstrated to improve the ability to tolerate anxiety while waiting for an invasive examination, such as an oral biopsy. With the present study, the authors aspire to attract attention to the possible role of graphic novel in dealing with patient anxiety, thereby becoming an invaluable tool to the medical practitioner in the field of oral medicine.
